# Relationship between Posterior Permanent Dentition Pattern and Radiographic Changes of the Mandibular Condyle

**DOI:** 10.3390/mps5060097

**Published:** 2022-12-04

**Authors:** Mahsa Esfehani, Marayam Tofangchiha, Neda Poorsayyah, Haniyeh Rahimi, Sarvin Kholafazadeh, Sina Radfar, Francesco Pagnoni, Rodolfo Reda, Luca Testarelli

**Affiliations:** 1 Department of Oral and Maxillofacial Medicine, School of Dentistry, Qazvin University of Medical Sciences, Qazvin 4199-15315, Iran; 2 Department of Oral and Maxillofacial Radiology, Dental Caries Prevention Research Center, Qazvin University of Medical Sciences, Qazvin 4199-15315, Iran; 3 Student Research Committee, Qazvin University of Medical Sciences, Qazvin 4199-15315, Iran; 4 Department of Orthodontics, Dental Caries Prevention Research Center, Qazvin University of Medical Sciences, Qazvin 4199-15315, Iran; 5 Department of Endodontics, Faculty of dentistry, Tabriz University of Medical Sciences, Tabriz 51666-14713, Iran; 6 Department of Oral and Maxillofacial Sciences, Sapienza University (University of Rome), 00161 Rome, Italy

**Keywords:** temporomandibular joint disorders, dentition, mandibular condyle

## Abstract

This study assessed the relationship between posterior permanent dentition and radiographic changes of the mandibular condyle. This descriptive, cross-sectional study was conducted on 300 panoramic radiographs of patients over 40 years of age (188 females and 112 males). Panoramic radiographs were evaluated for condylar changes such as flattening, subcortical sclerosis, subcortical cyst, erosion, osteophytes, and generalized sclerosis. Presence of muscle pain and temporomandibular joint (TMJ) pain and sounds, and history of TMJ trauma were also assessed. The occlusal scheme of posterior teeth was analyzed according to the Eichner’s index. The frequency of condylar changes was calculated in the right and left sides, and their association with posterior permanent dentition was analyzed by the Chi-square test (alpha = 0.05). The frequency of flattening, muscle pain, TMJ sounds, and erosion was 11.7%, 9.7%, 5.7%, and 3.7% in the right side, respectively. The frequency of flattening, muscle pain, erosion, and subcortical cyst was 12%, 9.3%, 5%, and 5% in the left side, respectively. The frequency of bilateral muscle pain, flattening, TMJ sounds, and TMJ pain was 18%, 16.7%, 11.7%, and 9.3%, respectively. Cases with TMJ trauma, generalized sclerosis, and osteophytes were few. According to the Eichner’s index, most patients with condylar changes had classes A and B, and a smaller percentage had class C. No significant difference was noted between healthy individuals and those with condylar changes regarding dentition patterns. No relationship existed between condylar changes and posterior permanent dentition pattern.

## 1. Introduction

The temporomandibular joint (TMJ) is a diarthrosis, better defined as a ginglymoarthrodial joint [1]. It has high adaptation capacity in normal function and also in response to functional stimuli [2]. The TMJ undergoes significant developmental changes from infancy until skeletal maturity. These changes continuously occur since birth and during the eruption process of primary and permanent teeth until skeletal maturation. On the other hand, the bone remodeling process normally continues after skeletal maturation and termination of development. Consequently, the skeletal structure of the joints acceptably adapts to the functional needs [3].

The mandibular condyle is part of the mandibular bone that also participates in the structure of the TMJ. It also serves as an area for regional adaptive growth while preserving its cartilage [4,5]. The condylar shape is categorized into several groups of flat, angled, round, convex, and concave. The morphological changes of the condyle occur due to remodeling, diseases, trauma, hormonal disorders, and radiotherapy [6]. Genetic factors and age also play a role in the shape and size of the condyles [7]. Condylar remodeling is a physiological process aiming to enhance the adaptation of the TMJ structure to meet the functional needs. This process is based on the correlation between the mechanical loads applied to the TMJ and adaptive capabilities of the condyle. The primary remodeling changes appear to be progressive and start with thickening of the soft articular tissue. These changes mainly occur on the surface of the condyle, and the TMJ articular surface and disc often remain unchanged or experience small changes [8]. The condylar cartilage adaptive remodeling occurs through a biomolecular pathway which starts by chondrogenesis and is finalized by osteogenesis. In the process of condylar adaptation, external stimuli such as condylar repositioning activate chondrogenesis. Such stimuli induce the differentiation of mesenchymal cells present in the articular layer of cartilage to chondrocytes that proliferate and turn into hypertrophic cells. The regulatory growth factors that adjust and monitor the phenotypic conversion of chondrocytes in the process of chondrogenesis are upregulated during the adaptive remodeling process to facilitate the transition of chondrogenesis to osteogenesis. In this process, hypertrophic chondrocytes and matrices are degraded and replaced with bone. This transition is sustained by an increase in neovascularization, which attracts osteoblasts and leads to subsequent new bone formation beneath the degraded cartilage [9].

**Figure 1:**
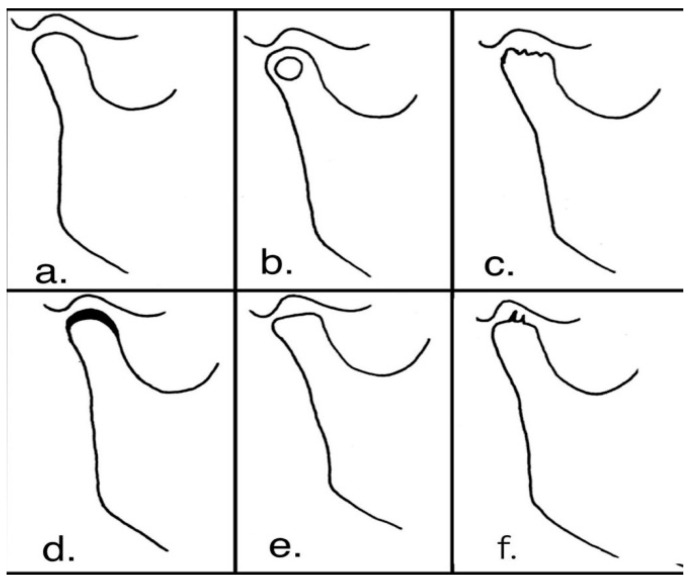
Condylar changes. (**a**) Normal condyle. (**b**) Subcortical cyst. (**c**) Erosion. (**d**) Sclerosis. (**e**) Flattening. (**f**) Osteophytes.

In hard articular tissue, the primary bone is gradually replaced with the secondary bone in response to changes in articular function. In some cases, bone remodeling occurs in the outer surface along with changes in the shape and dimensions of the joint (mainly in the condylar head), which is also detectable on radiographs [10,11]. Condylar changes can occur in the form of flattening, osteophytes, concavity, erosion, sclerosis, and subcortical cyst, and can be easily detected on panoramic radiographs [12] (Figure 1).

In such cases, since patients do not have any clinical symptoms, they do not seek any treatment. Additionally, the condyle has high potential for regeneration, which is helpful in orthodontic and surgical treatments.

A correlation has been documented between some clinical problems of the TMJ and radiographic findings [13]. Diagnostic imaging modalities for the TMJ include arthrography, computed tomography, magnetic resonance imaging, and plain radiography. Cone beam computed tomography (CBCT) is a 3D imaging modality with high diagnostic value for evaluation of bony structures of the TMJ [14]. However, CBCT has shortcomings such as higher image noise, and higher patient radiation than conventional radiography. CBCT, with a large field of view, has a seven times higher patient radiation dose than panoramic radiography [15]. In general, conventional radiographic modalities are always considered as a safer and more appropriate diagnostic tool for initial evaluation of patients. CBCT can serve as a suitable secondary option when a conventional radiograph is not sufficient for precise assessment of the main concern [16]. Low patient radiation dose, TMJ imaging, observation of both condyles on one radiograph, and providing valuable information about all teeth and other parts of the jaw are among the advantages of panoramic radiography. It is also one of the most commonly used imaging techniques by dental clinicians [17,18]. However, it may cause condylar distortion [19].

Assessment of the correlation of radiographic changes of the condyle and the occlusal scheme is important to find the related factors and strategies to prevent or treat them. Accordingly, this study aimed to assess the relationship between posterior permanent dentition and radiographic changes of the condyle.

## 2. Materials and Methods

This descriptive cross-sectional study was conducted on panoramic radiographs of patients over 40 years of age presenting to an oral and maxillofacial radiology clinic. The study protocol was approved by the ethics committee of Qazvin University of Medical Sciences (IR.QUMS.REC.1399.189).

The sample size was calculated to be 240 panoramic radiographs according to a study by Nah [20] assuming alpha = 0.05, and d = 0.06. To increase the accuracy of the results, the sample size was increased to 300 radiographs. Panoramic radiographs obtained for diagnostic or therapeutic purposes not related to this study were selected by convenience sampling.

The inclusion criteria were panoramic radiographs of dentate patients with no history of orthodontic treatment, craniofacial deformities, complete oral rehabilitation, trauma, maxillofacial surgery, TMJ ankylosis, or polyarthritis. Additionally, complete clinical information of patients was available in their records. Poor-quality radiographs, and patients with a history of TMJ pathologies, generalized arthritis, or continuous use of steroids or anti-inflammatory drugs as mentioned in patient records were excluded.

After obtaining written informed consent from the patients, they were questioned about any problem or pain in mastication, pain in the periauricular area, articular sounds, and mouth opening limitation. The radiographs were evaluated by two oral and maxillofacial radiologists, and radiographic changes of the condyles were evaluated according to the following criteria:

Articular surface flattening: Flattening of the round superficial contour of the condyle (flattening of the convexity and concavity in the outer articular borders).

Subcortical sclerosis: Increased thickness of cortical plate in areas under loading compared with unloaded adjacent areas.

Subcortical cyst: Presence of a cavity (radiolucency) beneath the articular surface which does not follow the normal pattern of the brain.

Surface erosion: Absence of integrity in the articular cortex due to resorption of the cortical plate of the articular surface.

Osteophytes: Marginal hypertrophy along with sclerotic borders and formation of superficial exophytic bony lesions.

Generalized sclerosis: Absence of a specific trabecular direction and inability to differentiate between the cortical plate and trabecular bone that has progressed towards the condylar head.

Loose joint body: Presence of specific calcified structures incompatible with the structure of the disc or bony structures of the joint.

The frequency of different condylar changes and TMJ disorders, and the pattern of posterior dentition according to the index described by Eichner et al. [21], were determined and recorded based on panoramic radiographic observations (Table 1).

Data were analyzed by SPSS version 25. The correlation between condylar changes and TMJ disorders with different patterns of posterior dentition was analyzed by the Chi-square test. Level of significance was set at 0.05.

## 3. Results

A total of 300 panoramic radiographs of 112 males (37.7%) and 188 females (62.7%) were evaluated in this study. Table 2 presents the frequency of condylar changes detected on panoramic radiographs according to the Eichner’s index [21].

No significant difference was noted between male and female patients or different age groups regarding the frequency of radiographic changes of the condyle and pattern of posterior dentition according to the Eichner’s index (*p* > 0.05).

## 4. Discussion

The TMJ is a synovial joint with unique properties in responding to environmental stimuli, surgical procedures, and trauma from occlusion. This joint undergoes adaptive changes since birth which continue after complete skeletal maturity. In skeletal part of the joint, remodeling occurs by gradual replacement of the primary bone with secondary bone in response to changes in function of the joint [22]. Morphological changes of the TMJ often include flattening, Ely cyst, osteophytes, sclerosis, and erosion, which can be easily detected on panoramic radiographs [23]. Accordingly, this study aimed to assess the relationship between posterior permanent dentition and radiographic changes of the condyle. This study used panoramic radiographs of patients due to their easier availability and according to studies by Mathew et al. [23] and Nelke et al. [24].

The frequency of flattening, muscle pain, TMJ sounds, and erosion was 11.7%, 9.7%, 5.7%, and 3.7% in the right side, respectively. The frequency of flattening, muscle pain, erosion, and subcortical cyst was 12%, 9.3%, 5%, and 5% in the left side, respectively. The frequency of bilateral muscle pain, flattening, TMJ sounds, and TMJ pain was 18%, 16.7%, 11.7%, and 9.3%, respectively. Cases with TMJ trauma, generalized sclerosis, and osteophytes were few. In the present study, no significant difference was noted between male and female patients or different age groups regarding the frequency of skeletal changes of the condyle and pattern of posterior dentition according to the Eichner’s index (*p* > 0.05).

Görürgöz et al. [25] analyzed the relationship between the articular eminence inclination, height, and shape, and degenerative condylar changes using CBCT. They found significant correlations between gender and age with degenerative condylar changes, which was different from the present results, and may be due to differences in the type of radiographic modality used for the assessment of joint changes.

Merigue et al. [26] performed tomographic evaluation of the TMJ in patients with malocclusion. They investigated condylar concentricity and morphology, and their association with Angle’s class I and II malocclusions. They did not find any significant association between the groups and condylar characteristics.

In a previous study on the effect of parafunctional habits on the condyles, it was observed that all patients with parafunctional habits had dissimilar condyles. The design of the present study did not allow for this factor to be assessed [27].

In a study by Jalalian and Alaei [28], Ely cyst was reported in 0.5%, and flattening was noted in 49.5% of the patients. Additionally, the prevalence of erosion, osteophytes, and sclerosis was 16.2%, 10.1%, and 10.0%, respectively. Their results were somewhat in line with the present findings. Nah et al. [20] evaluated the CBCT scans of 440 TMJs of 220 patients and reported that changes in posterior position of the condyle in the sagittal plane (68%), sclerosis (30.2%), articular surface flattening (25.5%), articular osteophytes (8%), and subcortical cysts (5.5%) were the most common findings. They also showed that 27% of the joints had signs of skeletal remodeling changes of the condyle. Their observations were different from the present findings, which may be due to the use of CBCT. Dos Anjos Pontual et al. [29] used CBCT and reported that flattening and osteophytes were the most common changes. The same results were reported by Guler et al. [30] using magnetic resonance imaging. Alexiou et al. [7] used CBCT to assess age-related changes of the condyle and reported that erosion, flattening, and osteophytes were the most common radiographic findings. Campos et al. [31] used magnetic resonance imaging and reported that osteophytes and erosion were the most common radiographic findings. Additionally, Talaat et al. [32] reported that osteophytes and flattening of the condylar surface were the most common skeletal changes of the TMJ. Their results were somehow in line with the present results. High prevalence of flattening in their study may be due to the fact that flattening is a type of adaptive change [33]. It may be a primary change in response to a progressive disease [34], or a secondary degenerative change in response to internal derangements [35]. Additionally, flattening is a degenerative change due to excessive loading of the TMJ and may be related to involvement of the masseter and temporalis muscles [36,37]. On the other hand, osteophytes occur in advanced degenerative changes and when the condyle wants to adapt to articular changes. Osteophytes appear to stabilize and widen the surface to neutralize the effects of excessive loading on newly formed cartilaginous areas. Erosion is the primary phase of degenerative changes of bone, and its presence indicates that the TMJ is not stable, and the skeletal surface alterations would probably change the occlusion [37,38,39].

De Melo et al. [40] evaluated skeletal changes in symptomatic young patients and reported condylar hypoplasia in 23.52%, condylar erosion in 16.66%, and osteophytes in 8.82% of the patients. Variations in the prevalence rates of abnormal radiographic findings reported in the literature may be due to selection of different age ranges of patients, sample size, radiographic modalities, and interpretation of radiographs. Additionally, it should be noted that temporomandibular disorders and condylar changes are multifactorial, and a number of psychological factors including stress play a role in their development [41]. The possible correlation between psychological conditions and temporomandibular disorders should be investigated in future studies.

In the present study, the majority of patients showing condylar changes had Eichner’s classes A (3, 2 or 1 occlusal contacts in 4 supporting zones) and B (3, 2 or 1 supporting zones in anterior teeth), and a smaller percentage had class C (no supporting zone in the remaining teeth) [21]. However, no significant difference was noted in dentition patterns between healthy individuals and those with condylar changes in the right, left, or both sides. In the study by Jalalian and Alaei [28], mandibular condylar changes in group A had a lower frequency than groups B and C. They added that temporomandibular disorders (TMDs) had no significant effect on condylar remodeling changes, and occlusion and edentulism had no direct correlation with remodeling changes either, which were similar to the present findings. Hiltunen et al. [42] found no association between occlusal interferences and condylar changes, which was in line with the present findings. However, some other studies showed significant correlations between condylar remodeling changes and dentition status according to Eichner’s patterns [7,43,44,45,46].

The main limitation of this study was using panoramic radiographs instead of CBCT scans. Panoramic radiographs are routinely requested by dental clinicians and since the aim of this study was to assess the correlation of posterior permanent dentition and condylar changes, an adequate number of CBCT scans was required for each dentition group; however, eligible CBCT scans visualizing both condyles and different types of posterior dentition were hard to find. Since selection of an appropriate size of field of view is critical to eliminate unwanted exposure [47], many available CBCT scans do not image both condyles. The selection of panoramic radiographs of TMD patients and healthy controls from one single center was another limitation of this study, which limits the generalizability of the results. Additionally, due to the cross-sectional design of the study and the ongoing COVID-19 pandemic, recall of patients and their clinical examination could not be performed, which was another limitation. Future studies are required using more advanced modalities such as CBCT. Additionally, systematic reviews for assessment of the prevalence of condylar changes in TMD patients and healthy controls with different classes of malocclusion can provide valuable information.

## 5. Conclusions

Flattening and muscle pain had the highest frequency in our study population. No relationship was found between condylar changes and permanent posterior dentition pattern. According to the Eichner’s index, most patients with condylar changes had classes A and B. No significant difference was noted between male and female patients or different age groups regarding the frequency of skeletal changes of the condyle and pattern of posterior dentition according to the Eichner’s index.

## Figures and Tables

**Table 1:** Pattern of posterior dentition according to the Eichner’s index.

Code	Definition
A1	4 occlusal supporting zones in presence of all teeth
A2	4 occlusal supporting zones despite some missing teeth in one dental arch
A3	4 occlusal supporting zones despite some missing teeth in both dental arches
B1	3 occlusal supporting zones
B2	2 occlusal supporting zones
B3	1 occlusal supporting zone
B4	Opposing contact in anterior teeth with no supporting zone
C1	0 supporting zone. Teeth in both arches are not in contact
C2	0 supporting zone. One arch is edentulous
C3	0 supporting zone. Both arches are edentulous

**Table 2:** Frequency of condylar changes detected on panoramic radiographs based on the Eichner’s class.

Skeletal Changes	Grouping	Number	Highest Frequency	Percentage
Muscle pain	Sound	189	A1	19.6%
	Right side	29	A1–A3	24.2%
	Left side	28	B1	21.4%
	Both sides	54	B1	22.2%
Joint sounds	Sound	236	A1	17.8%
	Right side	17	A3	29.4%
	Left side	12	B1	25.0%
	Both sides	35	A2	22.9%
Joint pain	Sound	257	A1	17.1%
	Right side	10	A1–A3	30%–30%
	Left side	5	B1	40%
	Both sides	28	A3	28.6%
Joint trauma	Sound	297	A1	18.2%
	Right side	0	0	0
	Left side	2	A2–B4	50%–50%
	Both sides	1	A2	100%
Generalized sclerosis	Sound	299	A1	17.7%
	Right side	1	A1	100%
	Left side	0	0	0
	Both sides	0	0	0
Osteophytes	Sound	296	A1	18.2%
	Right side	2	A3–B1	50%–50%
	Left side	1	A3	100%
	Both sides	1	A3	100%
Erosion	Sound	270	A1	17.8%
	Right side	11	B1–B2	27.3%–27.3%
	Left side	15	A1	26.7%
	Both sides	4	B1	75%
Subcortical cyst	Sound	262	A1–B1	17.2%
	Right side	6	B4	83.3%
	Left side	15	B1	26.7%
	Both sides	17	A1	35.3%
Subcortical sclerosis	Sound	285	A1	18.2%
	Right side	6	A2–B2–B4	33.3%
	Left side	4	B1–B2–C2–C3	25%
	Both sides	5	A1	40%
Flattening	Sound	179	A1	19%
	Right side	35	A1	34.3%
	Left side	36	B2	25%
	Both sides	50	A2	20%

## Data Availability

Not applicable.
